# Novel Use of Capture-Recapture Methods to Estimate Completeness of Contact Tracing during an Ebola Outbreak, Democratic Republic of the Congo, 2018–2020

**DOI:** 10.3201/eid2712.204958

**Published:** 2021-12

**Authors:** Jonathan A. Polonsky, Dankmar Böhning, Mory Keita, Steve Ahuka-Mundeke, Justus Nsio-Mbeta, Aaron Aruna Abedi, Mathias Mossoko, Janne Estill, Olivia Keiser, Laurent Kaiser, Zabulon Yoti, Patarawan Sangnawakij, Rattana Lerdsuwansri, Victor J. Del Rio Vilas

**Affiliations:** World Health Organization, Geneva, Switzerland (J.A. Polonsky);; Institute of Global Health, University of Geneva, Geneva (J.A. Polonsky, M. Keita, J. Estill, O. Keiser, L. Kaiser);; University of Southampton, Southampton, UK (D. Böhning);; World Health Organization Regional Office for Africa, Brazzaville, Republic of the Congo (M. Keita, Z. Yoti);; Institut National de Recherche Biomédicale, Kinshasa, Democratic Republic of the Congo (S. Ahuka-Mundeke, M. Mossoko);; Ministere de la Santé Publique, Kinshasa (J. Nsio-Mbeta, A. Aruna Abedi);; Thammasat University, Bangkok, Thailand (P. Sangnawakij, R. Lerdsuwansri);; World Health Organization South East Asia Regional Office, New Delhi, India (V.J. Del Rio Vilas)

**Keywords:** Ebola, Ebolavirus, viruses, contact tracing, disease outbreaks, Democratic Republic of the Congo, capture-recapture, zoonoses

## Abstract

Despite its critical role in containing outbreaks, the efficacy of contact tracing, measured as the sensitivity of case detection, remains an elusive metric. We estimated the sensitivity of contact tracing by applying unilist capture-recapture methods on data from the 2018–2020 outbreak of Ebola virus disease in the Democratic Republic of the Congo. To compute sensitivity, we applied different distributional assumptions to the zero-truncated count data to estimate the number of unobserved case-patients with any contacts and infected contacts. Geometric distributions were the best-fitting models. Our results indicate that contact tracing efforts identified almost all (n = 792, 99%) of case-patients with any contacts but only half (n = 207, 48%) of case-patients with infected contacts, suggesting that contact tracing efforts performed well at identifying contacts during the listing stage but performed poorly during the contact follow-up stage. We discuss extensions to our work and potential applications for the ongoing coronavirus pandemic.

Contact tracing is the process by which persons who are believed to have come into contact with a confirmed case patient with an infectious disease during the infectious period are located and checked for the presence of the infection or disease. Under traditional approaches, contact tracing involves 3 distinct steps: contact identification, in which potential contacts are identified through interview with the primary case-patient; contact listing, in which those identified contacts are listed and communication established with them; and contact follow-up, in which those listed contacts are monitored for presence of infection or onset of disease over a predefined period (*1*).

Because of its important role in case detection to monitor and curtail chains of transmission, contact tracing often forms part of the public health response to directly transmitted infectious diseases (*2*). Recently, contact tracing has received widespread attention because of its critical role in the response to outbreaks of diphtheria (*3*), Ebola virus disease (EVD) (*4*–*6*), and the ongoing coronavirus disease (COVID-19) pandemic (*7*,*8*).

During 2018–2020, the Democratic Republic of the Congo (DRC) experienced its 10th and largest EVD outbreak, the second largest ever experienced globally (*9*). EVD is a disease caused by viruses of the genus *Ebolavirus*, family *Filoviridae*. Zoonotic spillover events from the animal reservoir have led to large, explosive outbreaks in West and Central Africa in recent years (*9*–*12*). Owing to the high pathogenicity and virulence of Ebola virus, an elimination control strategy is always adopted, aiming to ensure that all case-patients are identified, isolated, and treated promptly after disease onset, thereby limiting the opportunity for onward community spread. Although contact tracing is a central pillar of control (*13*), no standardized methods have been established to assess a critical aspect of performance, its sensitivity (i.e., the ability to detect all contacts and secondary infections resulting from case-patients).

One approach to quantifying this metric is to employ capture-recapture (CRC) methods (*14*,*15*). Broadly, this family of methodologic approaches enables researchers to quantify any unit of interest missing from lists and subsequently estimate the sensitivity of the surveillance effort and the probability of detection. Although CRC has previously been used to estimate the number of unobserved cases of disease (*16*,*17*), such approaches typically rely on comparison of multiple lists, which are generally not available for contact lists. Therefore, we describe the application of a unilist capture-recapture approach (*15*) to quantifying the number of unobserved case-patients and contacts and describe their sociodemographic profile, helping to identify plausible risk factors that can be used to target limited resources at those unobserved case-patients most likely to generate onward transmission. More precisely, we aimed to address 2 questions, from which we can derive contact tracing sensitivity estimates: how many case-patients with any contacts did contact tracing miss, and how many case-patients with infected contacts did contact tracing miss?

## Materials and Methods

### Study Participants

We included all confirmed and probable EVD case-patients and contacts (classified according to standardized case definitions [*18*,*19*]) identified in Beni Health Zone, DRC, during July 31, 2018–April 26, 2020. Case-patients were principally detected through 3 identification mechanisms: passive detection at healthcare facilities from persons manifesting symptoms consistent with EVD, house-to-house active case-finding by community health workers, and tracing the contacts of EVD case-patients. Contact tracing was coordinated by the DRC Ministry of Public Health, with support from the World Health Organization, and conducted by locally recruited teams of contact-tracers. Upon detection of a case, efforts to identify and list the case-patient’s contacts were undertaken.

For case-patients, our data contain basic information on sociodemographic characteristics (e.g., age, sex, and DRC Health Area of residence) and dates of disease onset and isolation. For contacts, our data contain similar sociodemographic information and information on the daily follow-up and final status of the contact (either “completed the 21 days follow-up,” “confirmed as EVD case-patient,” “lost to follow-up,” “never seen,” or “died during follow-up”). Contacts recorded as “confirmed as EVD case-patients” were those identified by the contact tracing teams during the course of their work. EVD was assumed to be the cause of death for contacts recorded as “died during follow-up” because of the short interval between their contact with an EVD case-patient and their death.

### Exploratory Data Analysis

We determined the distribution of case-patients according to age, sex, and timing of disease onset. We used the Wilcoxon test to explore differences in continuous variables and the χ^2^ test for categoric variables to determine the distribution of the number of contacts per case-patient between 2 distinct epidemic waves. Overdispersion (i.e., superspreading) in the offspring distribution of secondary case-patients arising from infectious persons may have profound effects on control strategies in low-resource settings (*20*,*21*), and we describe the extent of this phenomenon in 2 ways: first, by assessing the proportion of infectious persons linked to 80% of onward transmission using methods described by Endo et al. (*22*); and second, by estimating the dispersion parameter (*k*) using methods described by Althaus (*23*).

We used a multivariable logistic regression model to explore risk factors associated with loss to follow-up, in which previously successfully traced contacts (i.e., those identified, listed, and among whom follow-up has begun) become untraceable at some point during the 21-day follow-up period. In such instances, contacts unable to be traced for 3 consecutive days are recorded as having been lost to follow-up, and no further attempts at tracing are made. To explore characteristics of case-patients with infected contacts, we calculated the mean number of contacts, mean age, and sex ratio of case-patients with >1 listed contact (among whom we can be confident that at least a minimal investigation was conducted), according to 3 categories: those with no infected contacts identified, those with exactly 1 contact, and those with >2 contacts.

### CRC Modeling

**Figure 7:**
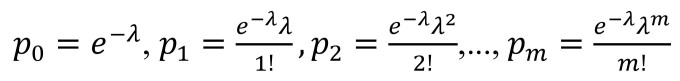
Equation

We classified the observed case-patients according to their number of listed contacts (either exactly 0 or >1 contact), further classifying this latter category according to the number of infected contacts observed (either exactly 0 or >1 contact). For each detected case, the contact tracing process generates a list of persons fitting the definition for a contact (Appendix), some of whom may themselves have been infected and will eventually become secondary case-patients. From this list, frequency distributions of case-patients with any listed contacts, and of case-patients with infected contacts, can be generated by first excluding (truncating) those case-patients with 0 contacts. For example, the data can be binned into the number of case-patients with exactly 1 contact (*f*_1_), 2 contacts (*f*_2_), and so on, to the number of case-patients with the maximum number of contacts (*f_m_*). Statistically, this process leads to a 0-truncated observed count distribution of case-patients with >1 contact. By applying a unilist CRC approach designed to estimate unobserved population sizes using the distribution of count data within single lists (*15*), we can infer *f*_0_, the number of unobserved case-patients with >1 contact. Associated with the observed frequencies (*f*_1_, *f*_2_,…, *f_m_*) and unobserved *f*_0_ are probabilities *p*_1_, *p_2_*,…, *p_m_* and *p*_0_ that inform the probability of identifying a case-patient with exactly 1, 2,…, *m* and 0 contacts, respectively. A conventional approach assumes that the frequencies arise from a discrete distribution such as the Poisson, where (Figure 7)

, , .., . 

**Figure 8:**
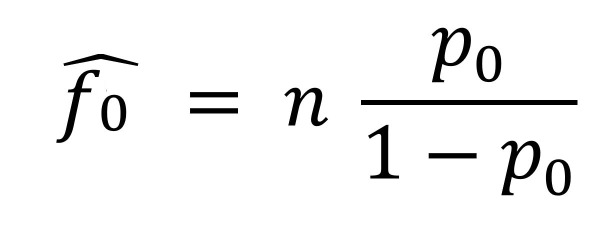
Equation

Other common distributions are the negative binomial and the geometric distribution. The geometric distribution has probabilities *p*_0_ = *p*, *p*_1_ = *p*(1 – *p*), *p*(1 – *p*)^2^…*p_m_* = *p*(1 – *p*)*^m^*, where *p* is a probability parameter. Poisson and geometric are special cases of the negative binomial distribution, which provides a flexible model family (Appendix). Because the observed distribution contains only positive numbers of contacts, we need to consider the associated zero-truncated distribution *p*_1_/(1 – *p*_0_), *p*_2_/(1 – *p*_0_),…*p_m_*/(1 – *p*_0_). In otherwords, we assume that the number of observed contacts among case-patients who actually had contacts follows a parametric distribution (although nonparametric approaches are possible [*15*,*24*,*25*]), find the best-fitting zero-truncated distribution of case-patients with >1 observed contact (we explore the zero-truncated Poisson, negative binomial, and geometric distributions [Appendix]), and use the estimated probability *p*_0_ of not observing a case-patient with contacts (calculated from the best-fitting distribution) to inform standard population estimators. We use the Horvitz–Thompson estimator to estimate *f*_0_, the unobserved number of case-patients (Figure 8):

where *n* is the number of observed case-patients with >1 observed contact and *p*_0_ is as previously defined. The Horvitz–Thompson estimator provides an unbiased estimate of *f*_0_, provided that *p*_0_ is correctly specified; hence, using a correctly-specified distribution for the number of observed contacts is important. We use maximum likelihood for model fitting, selecting the model with the smallest Akaike information criteria (AIC) and Bayesian information criteria (BIC) (Appendix).

To estimate 95% CIs, we use a parametric bootstrap, described as follows. Suppose that *N̂* is the estimated size of the (observed and unobserved) population under a fitted model. We generate *B* samples of size *N̂* using the fitted model and its estimated parameter or parameters. For each sample, all zeros are truncated and the size estimate *N̂_b_* computed, for each of the samples *b* = 1, …, *B.* We chose *B* = 10,000 to minimize bootstrap simulation random error. We constructed 95% CIs by using the 2.5th percentile of the distribution of *N̂_b_* as the lower end and the 97.5th percentile as the upper end.

## Results

### Exploratory Data Analysis

We identified 913 confirmed and 10 probable EVD case-patients in Beni Health Zone. The contact tracing process listed 80,556 contacts, of whom 6,375 were duplicates, having been listed as the contact of >1 case-patient, resulting in 74,181 contacts to trace. In discussion with contact tracing teams, duplicates were identified by matching name and residential location; for operational reasons, these persons were recorded as a contact of only the earliest-identified primary case-patient with whom they were associated. More than half of case-patients for whom sex and age information were available were women and girls (n = 515 [55.8%]); median age for all case-patients was 25 years (interquartile range [IQR] 13–38 years). Most contacts (64,545 [87.0%]) were successfully traced, leading to the detection of 396 secondary case-patients. The median delay between last contact with the primary case-patient and first contact by the contact tracing teams was 4 days (IQR 3–6 days).

**Figure 1:**
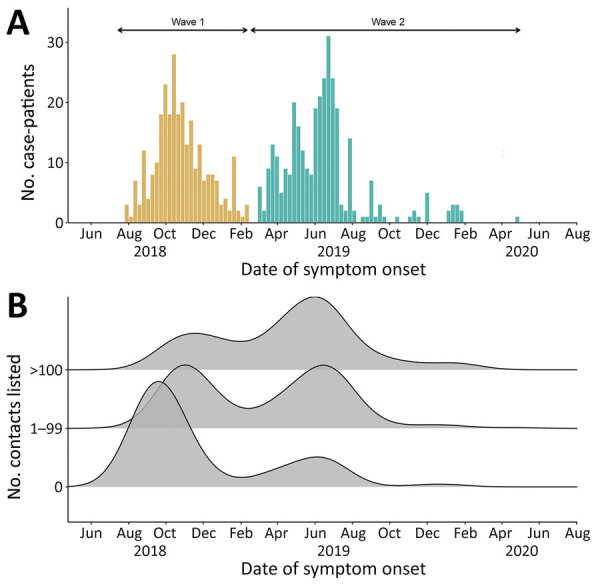
Epidemic curve and symptom onset dates among Ebola virus disease case-patients, Beni Health Zone, Democratic Republic of the Congo, July 31, 2018–April 26, 2020. A) Epidemic curve by date of symptom onset. Case-patients and contacts were divided into 2 epidemic waves, according to the date of symptom onset among case-patients (first wave, July 31, 2018–February 28, 2019; second wave, March 1, 2019–April 26, 2020). B) Distribution of dates of symptom onset among case-patients, by number of listed contacts. Data were smoothed by using a nonparametric (Gaussian) kernel-based estimate, with automatic bandwidth selection (37.6 days).

Disease onset dates spanned the period from July 31, 2018, to April 26, 2020, and was bimodally distributed, showing 2 waves that peaked in October 2018 and June 2019 (Figure 1, panel A). The second wave followed a period of insecurity in this conflict-affected area that severely hampered response activities (*26*).

**Figure 2:**
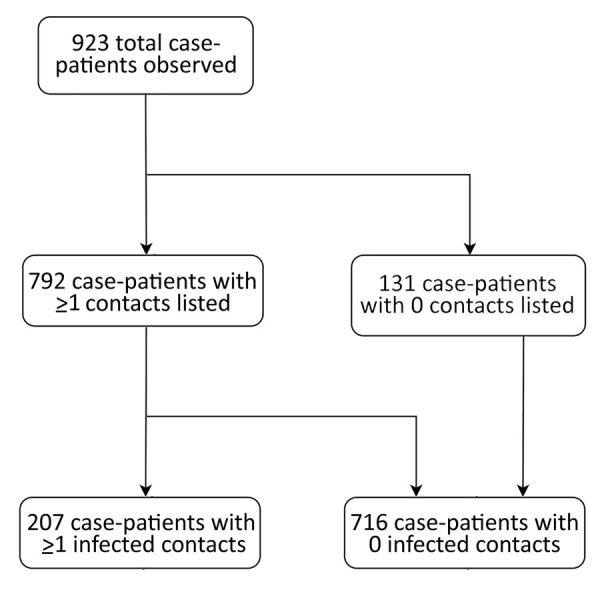
Flowchart showing breakdown of observed case-patients by number of listed and infected contacts among Ebola virus disease case-patients, Beni Health Zone, Democratic Republic of the Congo, July 31, 2018–April 26, 2020.

**Figure 3:**
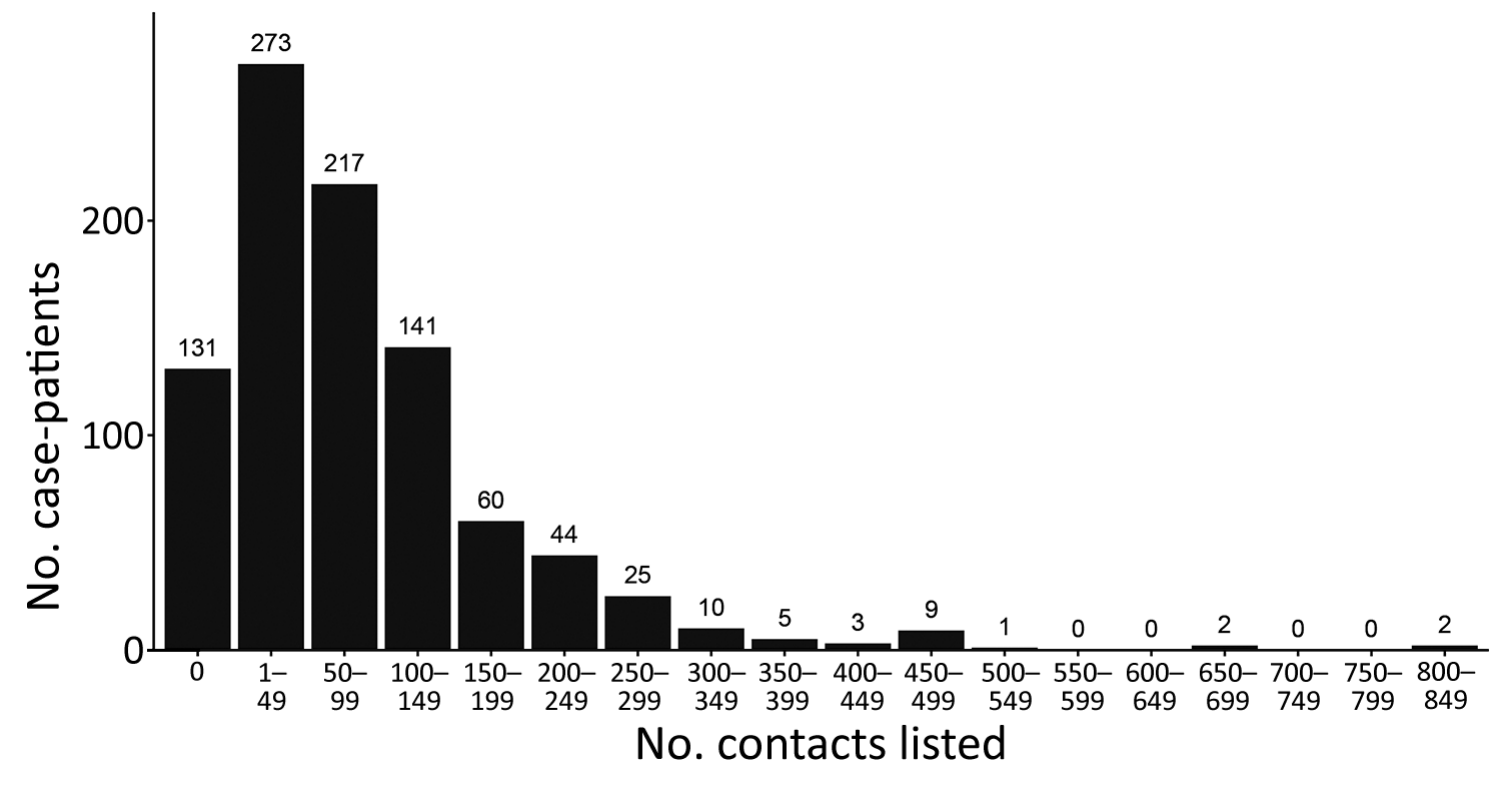
Frequency distribution of Ebola virus disease case-patients, by number of listed contacts, Beni Health Zone, Democratic Republic of the Congo, July 31, 2018–April 26, 2020.

The median number of contacts among all case-patients was 61 (IQR 18–120), but this number was significantly lower during the first wave than the second (34 vs. 80; p<0.001). Case-patients infected in the first wave were more likely to have 0 listed contacts than those in the second wave (31.3% vs. 9.6%; p<0.001 by χ^2^ test), and second-wave case-patients were more likely to have a large number (>100) of contacts (Figure 1, panel B). A total of 792 case-patients (85.8%) reported >1 contact (Figure 2, 3), among whom the median number of contacts was 74 (IQR 36–134) and the mean number of contacts was 102.

A total of 64,545 contacts (87.0%) were successfully traced, of whom 308 were confirmed as EVD case-patients and 88 died during follow-up. Therefore, the inferred total number of infected contacts was 396 (308 + 88), or 0.7% of the contacts successfully traced to completion of the follow-up period. Precise detail on the mechanism of identification of confirmed case-patients among contacts is not available; although we assume these infected contacts were identified by contact tracers during follow-up, the role of other surveillance activities cannot be excluded.

**Figure 4:**
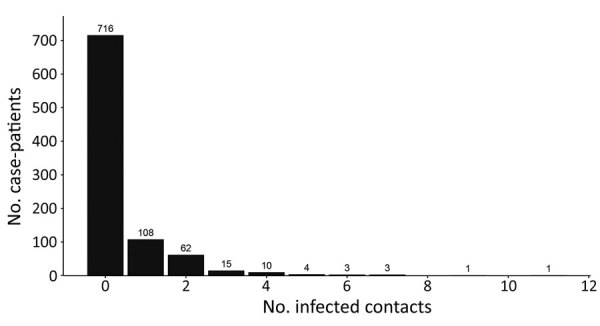
Frequency distribution of Ebola virus disease case-patients with infected contacts, by number of infected contacts, Beni Health Zone, Democratic Republic of the Congo, July 31, 2018–April 26, 2020.

We observed substantial overdispersion in the offspring distribution of secondary case-patients; 80% of onward transmission was linked to only 13.9% (95% CI 11.4%–16.2%) of primary case-patients, and all secondary case-patients concentrated among the contacts of 207 (22.4%) primary case-patients. Further, only 99 (10.7%) primary case-patients led to >1 secondary case-patient (Figure 2, 4). We estimated *k* as 0.27 (95% CI 0.20–0.33).

Male contacts had slightly (but statistically significantly) greater odds of being lost to follow-up (odds ratio [OR] 1.06, 95% CI 1.01–1.11) (Table 1). Contacts in older age groups had significantly greater odds of being lost to follow-up compared with contacts in the youngest age group (0–15 years). We observed the greatest effect among contacts >60 years of age (OR 1.65, 95% CI 1.47–1.86) and a marginally smaller effect among contacts 45–59 years of age (OR 1.55, 95% CI 1.43–1.69). Conversely, contacts traced during the second wave had lower odds of being lost to follow-up (OR 0.83, 95% CI 0.79–0.88).

**Table 1:** Multivariable logistic regression for predictors of loss to followup of contacts of Ebola virus disease case-patients, Beni Health Zone, Democratic Republic of the Congo, July 31, 2018–April 26, 2020*

Independent variable	No. contacts	Unadjusted			Adjusted	
		OR (95% CI)	p value		OR (95% CI)	p value
Sex						
F	41,349	Referent			Referent	
M	37,296	1.07 (1.02–1.12)	0.003		1.06 (1.01–1.11)	0.013
Age group, y						
0–14	20,616	Referent			Referent	
15–29	26,142	1.18 (1.11–1.25)	<0.001		1.19 (1.12–1.27)	<0.001
30–44	17,665	1.16 (1.09–1.24)	<0.001		1.18 (1.10–1.26)	<0.001
45–59	6,157	1.56 (1.43–1.70)	<0.001		1.55 (1.43–1.69)	<0.001
>60	2,599	1.64 (1.46–1.84)	<0.001		1.65 (1.47–1.86)	<0.001
Epidemic wave†						
First wave	14,374	Referent			Referent	
Second wave	66,182	0.85 (0.81–0.90)	<0.001		0.83 (0.79–0.88)	<0.001

*OR, odds ratio. †Contacts were divided into 2 epidemic waves according to the date of symptom onset of their associated primary case-patient (first wave, July 31, 2018–February 28, 2019; second wave, March 1, 2019–April 26, 2020).

### CRC Modeling

#### Completeness of Contract Tracing for Case-Patients with >1 Listed Contact

**Figure 5:**
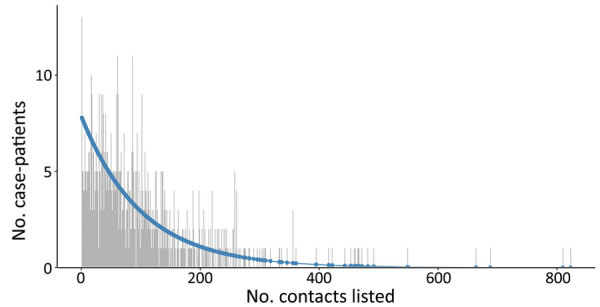
Observed (gray) and fitted (geometric; blue) zero-truncated distribution of the total number of contacts for case-patients with >1 contact listed, Beni Health Zone, Democratic Republic of the Congo, July 31, 2018–April 26, 2020..

**Figure 9:**
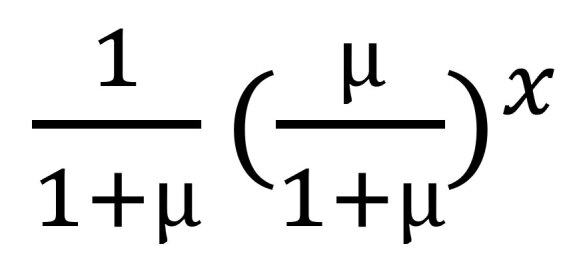
Equation

**Figure 10:**
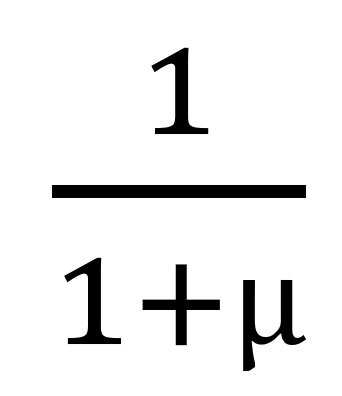
Equation

Among case-patients with >1 contact listed, the best-fitting distribution of the count of case-patients with any contacts was given by the zero-truncated geometric model, which produced the lowest AIC and BIC (Appendix Table 1). This distribution was very long-tailed (Figure 5), indicating that most case-patients with contacts were successfully detected, given that with an increasing mean of any count distribution, the probability for a zero count becomes smaller. This pattern is observed from the expression of the geometric distribution, described previously, where for *x* = 0 (i.e., the zero count), its estimated probability *p*_0_ resolves the equation (Figure 9)

 to return (Figure 10)

, where µ is the mean of the geometric model; the larger the mean, the smaller the probability of *x* = 0.

We estimated *p*_0_ (the unobserved number of case-patients with any contacts) = 8 (95% CI = 8–10), where sample size (*n*) was 792 and *p*_0_ was found as 0.01. The sensitivity of contact tracing to detect case-patients with any contacts was therefore 792/(792 + 8) = 0.99% (95% CI 0.99%–0.99%). We observed no difference in sensitivity by epidemic wave (wave 1 = 0.99% [95% CI 0.99%–0.99%]; wave 2 = 0.99 [95% CI 0.99–0.99]).

#### Completeness of Contact Tracing for Case-Patients with Infected Contacts

**Figure 6:**
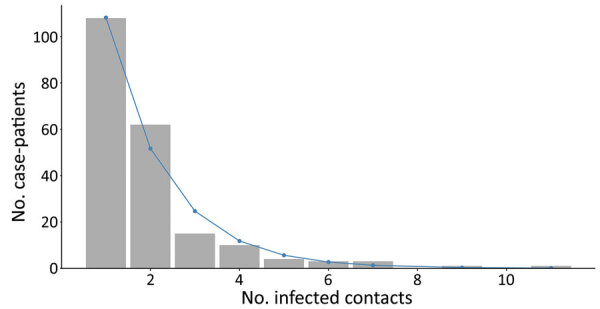
Observed (gray) and fitted (geometric; blue) zero-truncated distribution of the total number of infected contacts for case-patients with >1 infected contact listed, Beni Health Zone, Democratic Republic of the Congo, July 31, 2018–April 26, 2020.

Among case-patients with infected contacts, the best-fitting distribution of the count of case-patients with infected contacts was again given by the zero-truncated geometric model, which produced the lowest AIC and BIC (Appendix Table 1). This distribution is concentrated on the lower counts from 1 to 4 (Figure 6), indicating that a substantial proportion of case-patients with infected contacts may not have been detected.

We estimated *f*_0_ (the unobserved number of case-patients with infected contacts) = 227 (95% CI 171–241), where sample size (*n*) was 207 and *p*_0_ was found as 0.52. The sensitivity of contact tracing to detect case-patients with infected contacts was therefore 207/(207 + 227) = 0.49% (95% CI 0.43%–0.55%). We observed a statistically significant difference in sensitivity by epidemic wave, with lower sensitivity during wave 1 (0.24% [95% CI = 0.11%–0.38%]) than during wave 2 (0.48% [95% CI = 0.40%–0.56%]). Among the 792 case-patients with >1 listed contact, those with 0 infected contacts had fewer contacts overall, were slightly older, and were slightly more likely to be women or girls compared with the other groups (Table 2).

**Table 2:** Distribution of Ebola virus disease case-patients, their median age and sex ratio, and mean total number of contacts, grouped by number of infected contacts, among case-patients with >1 listed contact, Beni Health Zone, Democratic Republic of the Congo, July 31, 2018–April 26, 2020

No. infected contacts	No. case-patients	Median age of case-patients, y	% Women and girls among case-patients	Mean (95% CI) total number of contacts
0	585	28.2	59.5	85.7 (79.1–92.4)
1	108	23.6	56.7	122 (102.0–141.0)
>2	99	25.6	54.6	174 (144.0–204.0)

## Discussion

Our findings suggest that contract tracing efforts were very successful at identifying case-patients with >1 contact but much less successful at identifying case-patients with contacts who later had EVD symptoms. This finding is unsurprising, given that the investigation component (typically by interview with case-patients under treatment, their caregivers, or both) is easier to conduct than the tracing component (typically requiring daily visits to a large number of difficult-to-locate and mobile persons). This difference has important implications, because infected contacts contribute to ongoing chains of transmission when case investigation and contract tracing is inadequate; to prioritize scarce resources, control efforts should target those case-patients among whose contacts secondary infections arise (*20*,*21*,*27*). A high proportion of case-patients listed >1 contact (≈85%), compared with 27% during an EVD outbreak in Liberia (*28*) and 44% during an EVD outbreak in Sierra Leone (*27*), suggesting that lessons about enhancing the quality of contract tracing were learned from previous EVD outbreaks (*4*,*5*,*27*,*28*).

Case-patients with infected contacts had more contacts on average, which may result from 3 possible explanations. First, case-patients with more contacts are more likely to have >1 infected contact among these. Second, fewer overall listed contacts may be the result of poorly conducted case investigations. We found some evidence in support of this; the mean number of contacts increased as the epidemic progressed, indicating possible improvements in case investigation quality over time as staff became more accustomed to the procedure and community trust and engagement in the response improved (*29*). Third, case-patients with infected contacts may differ from other case-patients; in this study, such case-patients were younger and more likely to be men or boys, which are demographic factors previously shown to affect transmission of EVD and other diseases (*30*–*32*). Case-patients with more contacts have been shown to play a greater role in disease transmission and are more likely to have infected contacts (*33*,*34*). This tendency is particularly true of diseases that demonstrate heterogeneous transmission, including EVD and COVID-19, and our results suggest a high degree of overdispersion and superspreading, consistent with what has previously been reported during large EVD outbreaks (*23*). Overdispersion can lead to rapid expansion, particularly among hidden chains of transmission, and a promising area of research is to identify correlates of superspreading to better target limited resources for greatest impact. Previous research suggests that if highly infectious persons can be predictively identified and targeted, the efficiency of control can be greatly enhanced, such that focusing half of all control effort on the most infectious 20% of case-patients can improve effectiveness up to 3-fold (*20*,*21*).

Although estimating the number of unobserved case-patients with (infected) contacts is possible, identifying whether these case-patients have been misclassified as having 0 (infected) contacts or if they were undetected by the surveillance system in general is not possible. However, the greater probability of having 0 contacts listed during the first epidemic wave suggests substantial misclassification and suboptimal performance in the period during which surveillance activities were being established, as reported during previous EVD outbreaks (*4*,*27*,*28*). The sensitivity of contact tracing to detect case-patients with infected contacts was lower, and loss to follow-up greater, during the first epidemic wave, indicating quality improvements of this activity over time, either because the ability to conduct contact follow-up was hampered by the insecurity experienced during the first wave or because of greater familiarity with, and acceptance of, the process among contact tracing staff and the local population during later efforts.

Although the method we describe proposes a robust framework to assess the sensitivity of contact tracing, limitations include that no standard list of contacts against which to validate this method exists. However, the method itself has been validated to estimate actual population size in various other settings (*25*). The dataset does not permit the distinction between case-patients who were confirmed to have no contacts after a thorough case investigation and case-patients having no listed contacts because of no (or inadequate) case investigation. However, our method may help to identify the magnitude of the misclassification arising from this limitation. The inferences made are exclusively informed by the definition of case-patients as defined by contact tracing protocols; for example, our results would not inform the sensitivity of contact tracing as applied to asymptomatic EVD case-patients if these persons are not part of the testing strategy.

Differences in performance between contact tracers could result in strong heterogeneity in the count distribution, which might be detectable. For this reason, we applied Chao’s estimator (which allows for heterogeneity), and only if this was significantly different from the model-based estimate would we consider that an issue exists. In our results, we did not observe such a difference (Appendix Tables 2, 3). Finally, we have not adjusted for observed heterogeneity, such as age, sex, profession, geographic location of the case-patients, and delays in the contact tracing process. Further work is planned to incorporate such considerations.

In conclusion, contact tracing is crucial to the containing certain disease outbreaks. However, as with many surveillance activities, contact tracing has the potential to suffer reduced effectiveness from underreporting and poor sensitivity (*4*,*27*,*28*). The consequences of poor ascertainment and misclassification can be disastrous, potentially creating explosive expansion among hidden chains of transmission, particularly during containment and de-escalation phases.

We have described a novel application of CRC models to estimate a crucial yet elusive performance indicator of a key component of the public health response to epidemics, namely the sensitivity of contact tracing, as applied to a recent outbreak of EVD. The method demonstrated that most case-patients with any contacts were observed, suggesting that the case investigation component of contact tracing performed well, whereas less than half of case-patients with infected contacts were observed, suggesting that the contact follow-up component of contact tracing performed poorly in this setting. The approach described can be used to assess the sensitivity of contact tracing for any disease, including COVID-19, for which contact tracing has been identified as a crucial component of response activities.

Appendix Additional information about novel use of capture-recapture methods to estimate completeness of contact tracing during an Ebola outbreak, Democratic Republic of the Congo, 2018–2020.
