# Supplementary material for: Novel Use of Capture-Recapture Methods to Estimate Completeness of Contact Tracing during an Ebola Outbreak, Democratic Republic of the Congo, 2018–2020
Source: Emerg Infect Dis. 2021 Dec;27(12):3063–72. doi: 10.3201/eid2712.204958 (PMC8632194; doi:10.3201/eid2712.204958)
Supplement: Appendix — Additional information about novel use of capture-recapture methods to estimate completeness of contact tracing during an Ebola outbreak, Democratic Republic of the Congo, 2018–2020. [file 20-4958-Techapp-s1.pdf]

Article DOI: https://doi.org/10.3201/eid2712.204958

Novel Use of Capture-Recapture Methods to Estimate Completeness of Contact Tracing during an Ebola Outbreak, Democratic Republic of the Congo, 2018–2020

Appendix

Definition of Ebola contact

Any person having been exposed to a person with a suspected, probable, or confirmed case of Ebola in at least one of the following ways, provided that this exposure has taken place less than 21 days before the identification as a contact by surveillance teams:

- has slept in the same household with a case-patient
- has had direct physical contact with the case-patient (alive or dead) during the illness
- has had direct physical contact with the (dead) body of the case-patient at the funeral
- has touched his/her blood or body fluids during the illness
- has touched his/her clothes or linens
- has been breastfed by the patient (baby)

Details of models used for modeling zero-truncated distributions

The probability mass function (pmf) of a *Poisson distribution* is given by:

$$p_x = \frac{\exp(-\mu)\,\mu^x}{x!}$$

for x = 0,1,2,…, where μ is the parameter (mean) of the of the Poisson distribution.

The pmf of a *negative binomial distribution* is given by:

$$p_x = \frac{g(x+a)}{g(x+1)g(a)}(\frac{a}{\mu+a})^a(\frac{\mu}{\mu+a})^x$$

for x = 0,1,2,…, where $g(.)$ is the Gamma function, and μ (mean) and a are the parameters of the negative binomial distribution.

The pmf of a *geometric distribution* is given by:

$$p_x = p(1-p)^x$$

for x = 0,1,2,…, where p is the event parameter (between 0 and 1) in the geometric distribution. Note that

$$p = \frac{1}{\mu+1}$$

where μ is the mean of the geometric distribution.

The pmf's of the associated zero-truncated distributions for all of the three distributions above are

$$\frac{p_x}{1-p_0}$$

for x = 1,2,...

Note that the geometric arises as a special case of the negative binomial, where a = 1 and

$$p = \frac{1}{\mu+1}$$

which also corresponds to p_0.

Details of methods used to estimate the unobserved frequency of counts

We have also considered other estimation methods such as the nonparametric estimator of Chao and the Turing estimator to estimate the unobserved frequency when count of observations is zero (f_0):

MLE $\hat{f}_0 = \frac{n\hat{p}_0}{1-\hat{p}_0}$, where $\hat{p}_0 = P(X = 0) = \frac{1}{\mu+1}$

and μ = mean of geometric distribution.

Chao estimator $\hat{f}_0 = \frac{f_1^2}{f_2}$

Turing estimator $\hat{f}_0 = \frac{n\hat{p}_0}{1-\hat{p}_0}$, where $\hat{p}_0 = \sqrt{\frac{f_1}{s}}$, $s = f_1 + 2f_2 + \cdots + mf_m$,

and m is the largest observed count.

The population size N is estimated by $\hat{N} = \hat{f}_0 + n$, where n is the number of observed individuals. Note that we have based the analysis in our paper so far on the MLE. Appendix Table 2 presents the results for the indicator of interest for both the total population size as well as the number of unobserved individuals (case-patients). Appendix Table 3 provides uncertainty estimates for the values given in Appendix Table 3.

Appendix Table 1. Model performance for count of case-patients with any contacts and count of case-patients with *infected* contacts

	Case-patients with any contacts		Case-patients with infected contacts	
Model	AIC	BIC	AIC	BIC
Poisson	66635	66640	590	593
Negative binomial	8895	8905	551	558
Geometric	8900	8904	550	553

Appendix Table 2. Estimated population size and frequency of unobserved individuals using real datasets

Contact status	$\hat{N}$mle	$\hat{N}$chao	$\hat{N}$turing	$\hat{f}_0$mle	$\hat{f}_0$chao	$\hat{f}_0$turing
Contacts listed	800	826	802	8	34	10
Infected contacts and contacts died	434	395	433	227	188	226

Appendix Table 3. Estimated population size, unobserved frequency, and the 95% confidence interval using parametric bootstrap (pb) and bootstrap percentile (bp) methods

	Bootstrap method for $\hat{N}$					
Contact status	$\hat{N}$mle	$\hat{N}$chao	$\hat{N}$turing	pb.CI.mle, bp.CI.mle	pb.CI.chao, bp.CI.chao	pb.CI.turing, bp.CI.turing
Contacts listed	799.87	799.36	799.73	799.49–800.25, 779.34–800.45	791.93–806.80, 793–828	797.90–801.55, 769.80–802.53
Infected contacts and contacts died	434.59	435.48	434.55	400.47–468.72, 390.81–491.46	365.29–505.68, 350.46–568.78	397.99–471.10, 385.97–495.90
	Bootstrap method for $\hat{f}_0$					
Contact status	f0mle	f0chao	f0turing	pb.CI.mle, bp.CI.mle	pb.CI.chao, bp.CI.chao	pb.CI.turing, bp.CI.turing
Contacts listed	7.87	7.36	7.73	7.49–8.25, 7.34–8.45	0–14.80, 1–36	5.90–9.55, 4.80–10.53
Infected contacts and contacts died	227.59	228.48	227.55	193.47–261.72, 183.81–284.46	158.29–298.68, 143.46–361.78	190.99–264.10, 178.97–288.90
